# Photonic Characterisation of Indium Tin Oxide as a Function of Deposition Conditions

**DOI:** 10.3390/nano13131990

**Published:** 2023-06-30

**Authors:** Samuel F. J. Blair, Joshua S. Male, Stuart A. Cavill, Christopher P. Reardon, Thomas F. Krauss

**Affiliations:** School of Physics, Engineering and Technology, University of York, York, YO10 5DD, UK

**Keywords:** indium tin oxide, Drude model, guided mode resonance, epsilon-near-zero material, transparent conducting oxides, nanomaterial

## Abstract

Indium tin oxide (ITO) has recently gained prominence as a photonic nanomaterial, for example, in modulators, tuneable metasurfaces and for epsilon-near-zero (ENZ) photonics. The optical properties of ITO are typically described by the Drude model and are strongly dependent on the deposition conditions. In the current literature, studies often make several assumptions to connect the optically measured material parameters to the electrical properties of ITO, which are not always clear, nor do they necessarily apply. Here, we present a comprehensive study of the structural, electrical, and optical properties of ITO and showed how they relate to the deposition conditions. We use guided mode resonances to determine the dispersion curves of the deposited material and relate these to structural and electrical measurements to extract all relevant material parameters. We demonstrate how the carrier density, mobility, plasma frequency, electron effective mass, and collision frequency vary as a function of deposition conditions, and that the high-frequency permittivity ($\epsilon_{\infty }$) can vary significantly from the value of $\epsilon_{\infty }$ = 3.9 that many papers simply assume to be a constant. The depth of analysis we demonstrate allows the findings to be easily extrapolated to the photonic characterisation of other transparent conducting oxides (TCOs), whilst providing a much-needed reference for the research area.

## 1. Introduction

Indium tin oxide (ITO) is a transparent conducting oxide (TCO), most commonly used as a transparent electrode in photovoltaic and touchscreen applications [1,2]. More recently, ITO has also gained popularity as a photonic material, especially for light modulation [3], use in tuneable metasurfaces [4,5,6] and efficient light-matter interaction in the epsilon-near-zero (ENZ) regime [7]. ITO can be deposited using a variety of techniques, such as pulsed laser deposition [8], direct current (DC) and radio frequency (RF) magnetron sputtering [9] and electron beam deposition [10]. It is well understood that the photonic properties of ITO are sensitive to small variations in deposition conditions, such as oxygen flow, operating pressure and the deposition method. We also note that the literature detailing these effects is somewhat ambiguous. For example, many authors assume literature values for mobility (μ), the high-frequency permittivity ($\epsilon_{\infty }$), the electron effective mass (m${}_{e}^{*}$) and the collision frequency (Γ) without querying the conditions for which these values were obtained, while it is understood that many of these parameters vary as a function of the deposition condition. Here, we highlight these variations and show how the deposition conditions can be adjusted to yield desired properties.

We start by considering the complex dielectric permittivity of ITO which can be described by the simplified Drude model (Equation (1)) that allows us to determine the real and imaginary permittivity components and their dispersion. Furthermore, the carrier density (N), mobility (μ) and conductivity (σ) are linked through Equation (3).

(1) $$\epsilon \left(\omega \right)=\epsilon_{\infty }-\frac{\omega_{p}^{2}}{\omega^{2}+i\Gamma \omega }$$

(2) $$\omega_{p}^{2}=\frac{Ne^{2}}{\epsilon_{0}m_{e}^{*}}$$

(3) $$N=\frac{\sigma }{\mu e}$$

Here, $\epsilon_{\infty }$ represents the high-frequency permittivity, ω represents the angular frequency, $\omega_{p}$ represents the plasma frequency, Γ represents the collision frequency (which is a proxy for the damping coefficient), $\epsilon_{0}$ represents the vacuum permittivity, m${}_{e}^{*}$ represents the electron effective mass and e represents the electron charge.

The dispersive nature of the Drude model allows us to visualise the shift of the plasma frequency ($\omega_{p}$) towards a shorter wavelength as a function of increasing carrier density. This effect moves the epsilon-near-zero (ENZ) region, which is the region where the real dielectric permittivity fluctuates between values of −1 and 1 [11] towards the optical regime. The ENZ region is popular because it affords more efficient light–matter interaction, due to the $\frac{\Delta \epsilon_{r}}{\epsilon_{0}}$ dependence of many linear and nonlinear effects. No matter which wavelength range is preferred for a given application, the Drude fit parameters, such as N, $\epsilon_{\infty }$, μ and Γ, all impact on the dispersion curves. Therefore, simply assuming these parameters to be constant, or making assumptions about them that are not actually true, may lead to significant errors in determining the material properties or interpreting the data. As a case in point, $\epsilon_{\infty }$ is often assumed to be constant at $\epsilon_{\infty }$ = 3.9 [12,13,14,15,16,17,18,19] independently of the deposition condition.

**Figure 1:**
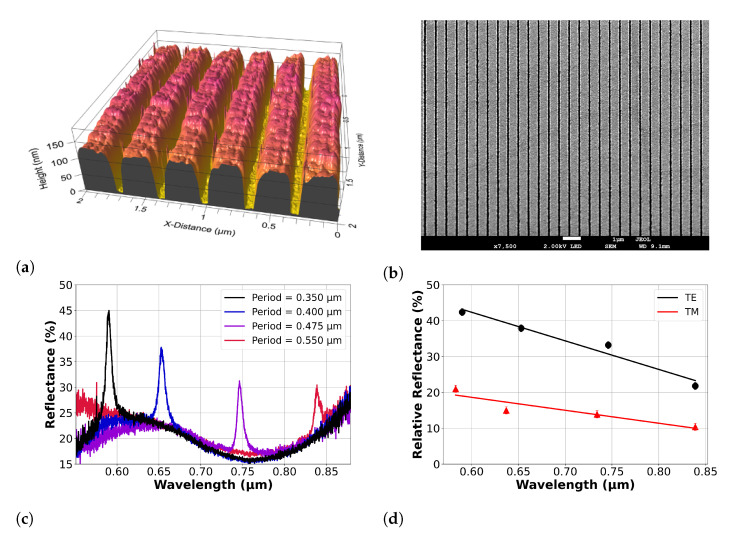
Structural and optical analysis methods used on ITO thin films with guided mode resonance grating structures written directly into the films. These are (**a**) AFM scan, (**b**) SEM image, (**c**) TM optical results for four gratings of different periods on a single film and (**d**) TE and TM relative amplitude values for increasing periods, displaying the effective loss increase with wavelength.

Here, we conduct a comprehensive analysis on a variety of ITO thin films deposited using a range of conditions to unambiguously determine the ITO dispersion properties and parameters. We achieve this by the fabrication of gratings that support guided mode resonances in the ITO thin films; the spectral position and amplitude of these resonances then allow us to accurately determine the real and imaginary dielectric constants. Furthermore, by fabricating structures directly into the films, the analysis incorporates the full dispersive properties of an actual device as it might be used in an application rather than that of an unstructured thin film alone. We vary the oxygen concentration during deposition and annealing to control the dispersive properties of the material. We then use a variety of techniques, such as atomic force microscopy (AFM) micrograph analysis (Figure 1a), scanning electron microscope (SEM) (Figure 1b), optical spectroscopy (Figure 1c), Hall measurements and X-ray diffraction (XRD) to characterise the films. Finally, a regressive analysis serves to determine a unique solution to the full dataset of N, $\epsilon_{\infty }$, μ, Γ, m${}_{e}^{*}$, $\omega_{p}$, $\epsilon_{r}$ and $\epsilon_{i}$.

## 2. Materials and Methods

ITO thin films were deposited on glass substrates via DC magnetron sputtering [20], using a 90:10 ITO target (In${}_{2}$O${}_{3}$(90%)/SnO${}_{2}\left(10\%\right)$). A constant Ar flow rate was used for all depositions, while the O${}_{2}$ flow rate was varied (0, 0.5, 1, 2.5, 3.5, 5, 7.5 standard cubic centimetres (SCCM)) to adjust the film conductivity. All samples were left to cool before nitrogen exposure, followed by annealing in an oxygen environment, aiding the crystallisation of ITO films that tend to be amorphous post-deposition. Sheet resistance values were taken using a four-point probe measurement, Hall measurements were used to determine mobility and XRD analysis to determine crystallinity. Finally, electron beam lithography (EBL) was used to write the guided mode resonance (GMR) grating patterns into the films, followed by reactive ion etching (RIE). Gratings were fabricated with a grating thickness of ∼150 nm and a filling factor of 0.7. The refractive index of the glass substrate was assumed to be a value of 1.47 [21].

AFM measurements (Figure 1a) and SEM micrographs (Figure 1b) were taken for each film, providing an accurate determination of the grating features. Optical spectroscopy data were then used to determine the wavelength of each grating resonance for both TE and TM polarisations. Example spectra are displayed in Figure 1c for four GMR gratings of differing periods on a single ITO film deposited with 20% oxygen flow during deposition. From the optical data, the refractive index and effective loss were determined by Fano fitting [22] and RCWA modelling. Here, the mode with the dominant electric field oriented along the grating grooves is defined as the transverse electric (TE) mode, and the mode with the dominant electric field perpendicular to the grating plane as the transverse magnetic (TM) mode. Figure 1d displays the relative amplitude (i.e., peak to trough) decrease with wavelength for the same sample shown in Figure 1c. The material loss can clearly be seen to increase with the wavelength by the decrease in resonance amplitude. Carrier densities were initially derived from the Hall probe measurements to allow an estimate of the dispersion curve of each film. Using a regression method, the dispersion curve was then fitted to the real and imaginary permittivity values generated from the GMR data to uniquely determine the values of each relevant parameter, i.e., the carrier density (N), electron effective mass (m${}_{e}^{*}$), high-frequency permittivity ($\epsilon_{\infty }$), plasma frequency ($\omega_{p}$) and collision frequency (Γ). More details of the experimental method are provided in the Supplementary Material, Part 1.

## 3. Results

### 3.1. ITO Conductivity

**Figure 2:**
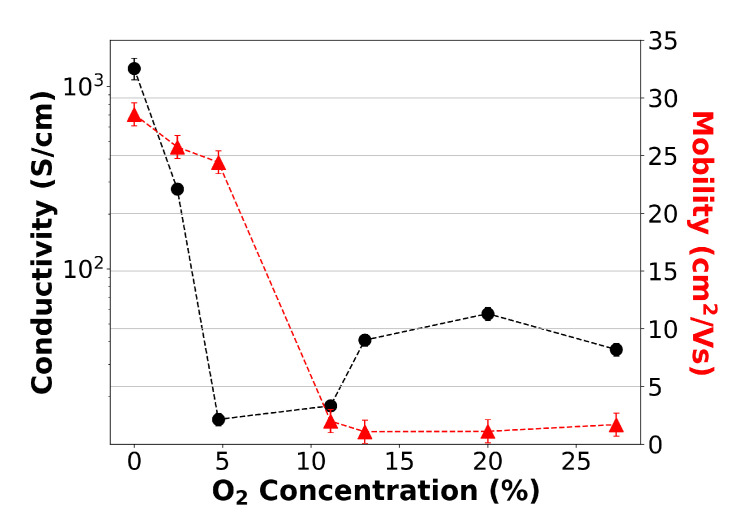
Oxygen concentration during deposition as a function of conductivity for several variations of oxygen flow. Conductivity measurements were taken after a standardised anneal. A dip in conductivity can be seen at 5%; however, the largest conductivity was measured at a 0% oxygen flow. Error bars are present but are of a small magnitude.

It is well understood that the free carriers in ITO arise from oxygen vacancies and Sn donors [23]. Oxygen vacancies are found to make the dominant contribution to the conductivity of ITO, contributing two free electrons, whilst the Sn${}^{+}$ ions donate only a single electron [24]. Therefore, we expect the conductivity to decrease with increasing oxygen flow [25,26] as the excess oxygen fills the oxygen vacancies. This dependency is indeed observed in the low-oxygen-flow regime, as seen in Figure 2, displaying conductivity and Hall probe electron mobility measurements. For a 0% oxygen flow during deposition, we obtained the highest conductivity at a value of (1.3 ± 0.2) ×$10{}^{3}$ S/cm. By introducing an external oxygen flow, the conductivity rapidly decreases, reaching a minimum value of 16 ± 1 S/cm for 5% flow. This trend then breaks down as the oxygen flow is further increased, and the conductivity increases again for a concentration of >10%. We associate this increase in conductivity with a higher number of substituted Sn ions with increasing oxygen flow, providing additional carrier contributions [27]. A saturation in the conductivity is then observed due to the equilibrium of the oxygen vacancy carrier contribution decrease and Sn ion carrier increase.

As clearly shown by Equation (3), carrier density and conductivity are directly connected to mobility. The dependence of the mobility on carrier density is a well-documented area in semiconducting devices [28,29]. However, this dependence becomes more complex in metal-oxides, involving aspects such as deposition method and the environment [30]. To further understand the material properties, we determined the mobility of each material using Hall-probe measurements (for detail, see Supplementary Material, Part 1). The results are shown as the red dashed line in Figure 2. We observe the singular trend that the mobility drops as a function of oxygen concentration. Since, intuitively, mobility can be understood as the measure of how quickly an electron can move through the semiconductor in the presence of an electric field, it is worth examining the crystallinity of the material; one would expect a highly crystalline material to exhibit high mobility, while a polycrystalline material, due to the presence of grain boundaries that impede transport, would be expected to exhibit a lower mobility [31,32]. As such, we hypothesise that the mobility measurements indicate a decreasing film crystallinity with increasing oxygen flow.

**Figure 3:**
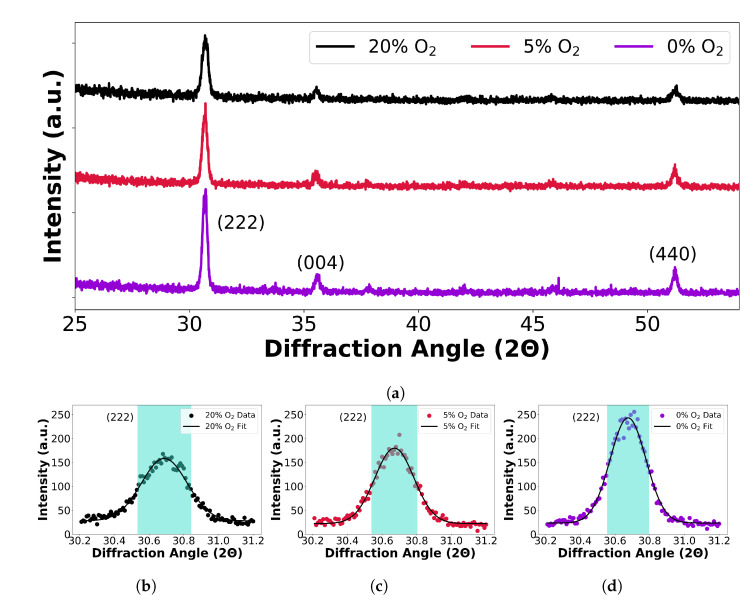
(**a**) XRD data for annealed ITO films of 0%, 5% and 20% oxygen flow during the deposition; (**b**–**d**) show exemplar FWHM extractions for the respective (222) orientation for 0%, 5% and 20% oxygen flow.

In order to verify this hypothesis, we examined the crystallinity of the samples by conducting XRD measurements. Three samples were analysed, namely annealed films for 0%, 5% and 20% oxygen flow during deposition. The results are shown in Figure 3, with the Miller indices indicating the observed diffracting planes of the Ia3 cubic space group. Peaks are visible for the (222), (004) and (440) planes, all of which match the literature Bragg angles for sputtered ITO [33]. The crystallinity, or degree of crystallisation, can be determined from the width of each diffraction peak; the narrower the peak, the more crystalline the sample and the larger the average grain size. This effect is mathematically expressed by the Scherrer equation [34] (Equation (4)), where δ is the mean size of a crystalline domain (which we approximate as the average grain size), λ is the wavelength of incident X-ray radiation, and Δλ is the full-width-half-maximum of the respective reflection and θ the Bragg angle. The crystallinity should directly correlate with mobility because mobility is higher in a pure monocrystal compared to a polycrystalline film with many grain boundaries.

(4) $$\delta =\frac{0.9\lambda }{\Delta \lambda cos\theta }$$

This is indeed what we observe, as the crystallinity decreases with increasing oxygen flow; the more oxygen is incorporated into the sample, the poorer the crystal becomes and the lower the mobility. Accordingly, the linewidth of the 5% oxygen sample is broader than that of the 0% oxygen sample, and that of the 20% sample is broader still. Hence, Table 1 shows average grain size data for each of the three ITO films of differing conductivity. The grain size is seen to decrease with increasing oxygen, i.e., the highest conductivity film (0%) possesses the highest average grain size. This finding aligns with the mobility trend in Figure 2, where the introduction of an external oxygen flow during deposition reduces the grain size of ITO films, decreasing the mobility.

It should be noted that an annealing step is crucial for achieving highly conductive ITO films. ITO is known to be amorphous post-deposition [24]; however, if annealed, it forms a polycrystalline structure. This is due to the relaxation of distorted bonds in the amorphous film and a reduction in impurities [24,35]. An annealing temperature of 500 °C with an oxygen flow of 1 × 10${}^{3}$ SCCM was found to provide the highest conductivity across all oxygen variations during deposition (Supplementary Material, Part 4). Temperatures lower or higher than this value decrease the conductivity. We note that ITO has a crystallisation temperature of approximately 400 °C [36]. It therefore makes sense that the optimal annealing temperature is somewhat above this value.

### 3.2. ITO Dispersion

**Figure 4:**
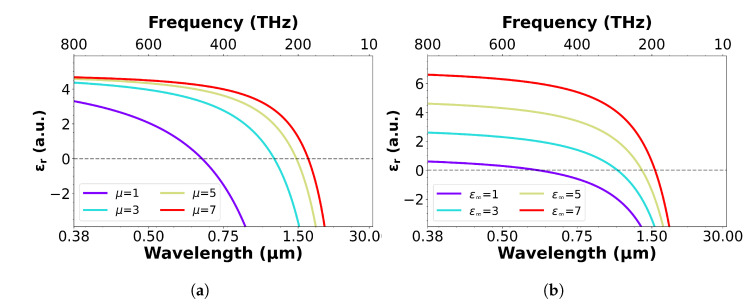
Simulated values of (**a**) increasing mobility ($\epsilon_{\infty }$ = 4.9 and m${}_{e}^{*}$ = 0.4 m${}_{e}$ kg) and (**b**) high-frequency permittivity (μ = 30 cm${}^{2}$/Vs and m${}_{e}^{*}$ = 0.4 m${}_{e}$ kg) for an ITO film of N = 3 × 10${}^{20}$ cm${}^{-3}$. The ENZ region can be seen to shift to higher wavelengths for increasing values on both plots.

To highlight the importance of the material parameters on the shape of the dispersion curve, Figure 4 displays how the μ and $\epsilon_{\infty }$ affect dispersion. Here, $\epsilon_{\infty }$ is plotted for a film deposited with 0% oxygen flow (N = 3 × 10${}^{20}$ cm${}^{-3}$). It can be seen that the changes in $\epsilon_{\infty }$ shift the y-intercept upwards with increasing values, also shifting the ENZ region to longer wavelengths. As this plot is for a single carrier density, it can clearly be seen that the true curve is at first ambiguous. Furthermore, the μ acts analogously to the carrier density, as changes in μ create carrier density variation. As such, the mobility plotted with a $\epsilon_{\infty }$ of 4.9 produces a shift in the ENZ region to larger wavelengths with increasing values, as well as significantly changing the gradient of the curve. Changes in mobility also have a stronger impact at shorter wavelengths. With high conductivity often being a desirable characteristic, there is indeed an incentive to experimentally determine the value of μ. Whilst the simulations in Figure 4 may seem trivial, it is surprising how many papers in the literature assume or fail to accurately obtain many of the relevant parameters. As such, a significant source of error may be introduced when attempting to determine the material properties.

**Figure 5:**
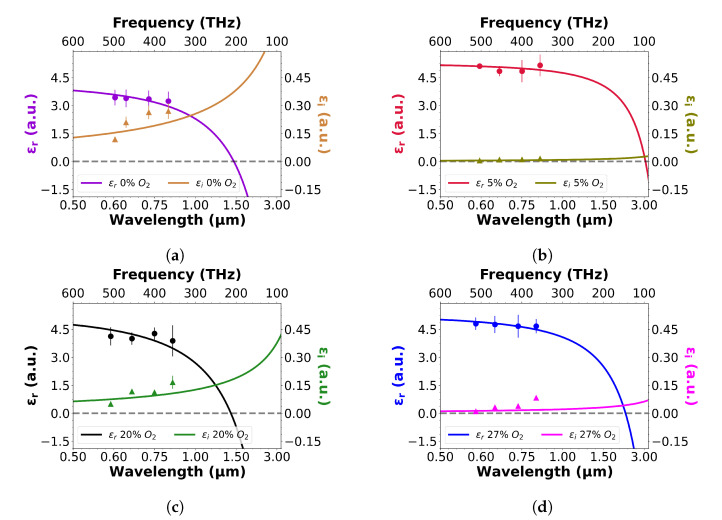
Real and imaginary dielectric permittivity as a function of wavelength (frequency) for ITO films deposited at: (**a**) 0%, (**b**) 5%, (**c**) 20% and (**d**) 27% oxygen concentration during deposition. Imaginary permittivity values are plotted for TE polarisation. See Supplementary Material, Part 9 for more detail, on the chosen wavelength regime.

Our dispersion curves are experimentally determined by matching the spectral positions of guided mode resonances for both TE and TM polarisation against simulations. As is well known, the effective index of a structure depends on polarisation, as differently polarised modes experience different boundary conditions that lead to a different field confinement. However, the refractive index at a given wavelength is an intrinsic material property, regardless of polarisation. Hence, by matching the conditions for both the TE and TM modes in simulation, an accurate determination of the refractive index and absorption loss can be made. By including the experimentally obtained mobilities and carrier densities, and by fitting multiple resonances at different wavelengths, we can determine the high-frequency permittivity ($\epsilon_{\infty }$), plasma frequency ($\omega_{p}$), collision frequency (Γ) and electron effective mass (m${}_{e}^{*}$) as fitting parameters with high confidence. Typically quoted electron effective mass results for ITO are in the range of 0.3 m${}_{e}$–0.6 m${}_{e}$ [37,38]; hence, we used this range in our regression analysis and found values between 0.42 m${}_{e}$ and 0.51 m${}_{e}$ (Table 2). The experimentally determined dispersion curves can be seen in Figure 5.

The discussion thus far has focused on the real part of the dispersive properties of ITO; clearly, the imaginary part is just as important for device applications. Here, a key parameter is Γ in Equation (1), which is often assumed to be a constant value at 180 THz [12,39]. In order to determine whether this value is correct or not, we need to experimentally measure $\epsilon_{i}$. As we are using device structures to determine the material parameters, the measured loss associated with the GMR resonances is a combination of scattering loss and absorption loss, with $\epsilon_{i}$ only representing the absorptive part. For the purpose of this study, we assumed the scattering loss to be constant for all films. Scattering loss arises from roughness and other fabrication imperfections which are constant for all samples, and while we recognise that there is a wavelength dependence, the variation in the scattering loss tends to be small over the wavelength range (Δλ = 600–850 nm) considered here.

We determined the loss from the amplitude of the guided mode resonance, amplitude being more sensitive to loss than the Q-factor, as comprehensively shown recently [40]. By determining the loss from the low conductivity samples, we can identify the scattering loss, as absorption is negligible in these. The same scattering loss was then added to all the other samples to determine the absorption loss from the resonance amplitude and expressed as $\epsilon_{i}$ in Figure 5.

## 4. Material Parameter Discussion

With all the parameters now determined, they are summarised in Table 2, which allows us to discuss some of the inter-dependencies.

### 4.1. Carrier Density, N

It is expected that the carrier density scales as the conductivity, although not linearly because of the additional impact of mobility according to Equation (3).

### 4.2. Plasma Frequency, $\omega_{p}$

As previously mentioned, the plasma frequency corresponds to the point where $\epsilon_{r}$ = 0 [41] (i.e., the ENZ regime). Hence, $\omega_{p}$ is expected to trend with film conductivity; the higher the carrier density, the more metallic the film, producing a shift in the plasma frequency to higher frequency values [42,43]. We find the plasma frequency to indeed trend linearly with conductivity and carrier density, according to Table 2.

### 4.3. Effective Mass, $m_{e}^{*}$

The effective mass is related to the real dielectric permittivity, as shown in Equations (1) and (2). With effective mass defined as a particle’s mass in response to external forces, one would expect the effective mass to be higher in a high carrier density film, as the increased number of carriers produces a larger potential. As such, a variation in the effective mass impacts on the real part of the dispersion curve, similarly but inversely to that of mobility, shifting the ENZ point to shorter wavelengths for decreasing values. This hypothesis is indeed the trend we observe, with $m_{e}^{*}$ being at a minimum value for the lowest conductivity film.

### 4.4. High-Frequency Permittivity, $\epsilon_{\infty }$

The high-frequency permittivity is the real permittivity at a short (ideally, zero) wavelengths and is determined by the bound carriers. Most authors assume $\epsilon_{\infty }$ to be a constant, but on reflection, this is clearly an oversimplification; take the case of silicon, where amorphous silicon tends to have a higher refractive index than crystalline silicon; in analogy, one should expect that the degree of crystallinity impacts on $\epsilon_{\infty }$. In fact, this is also what we observe; according to Table 1 and Table 2, $\epsilon_{\infty }$ scales inversely with the grain size; the sample with the largest crystal size is the 0% oxygen sample, which also has the lowest $\epsilon_{\infty }$. Hence, it is important to consider the variation of $\epsilon_{\infty }$ and not simply assume a fixed value.

### 4.5. Collision Frequency, Γ

The analysis also revealed variations in Γ for the different samples, albeit at the top end of the $10^{2}$ THz range. Again, given that many papers in the literature quote a value of 180 THz, we note that Γ does vary significantly as a function of the deposition condition. In particular, an inverse relationship between Γ and μ is apparent, in that a higher frequency of collisions (shorter time between collisions $\tau =\frac{1}{\Gamma }$) suggests a smaller electron mobility, as the electrons cannot move freely through the material due to the increased grain boundary scattering. Hence, using the grain boundary trend in Figure 3, one would expect a low conductivity film to possess a larger Γ, which is indeed what we observe in Table 2, with the 0% oxygen flow providing the lowest value for Γ. Furthermore, the findings suggest a greater degree of complexity, particularly for the low absorption loss films. In polycrystalline films, such as ITO, specific conditions like a small grain size relative to the mean free path can perturb the collision frequency trend [44,45].

It is thus evident that any of the Drude parameters individually shape the dispersion curve of ITO. Hence, it is stressed that, by tailoring the deposition and annealing conditions, one can determine, and in fact tune, an accurate dispersion curve and extract all relevant material parameters for ITO films of differing conductivity.

## 5. Discussion

The experimentally determined real and imaginary dispersion trends in Figure 5 are similar to those obtained by ITO ellipsometry measurements reported in the recent literature. For example, ITO films optimised for high conductivity via the oxygen flow during deposition tend to exhibit lower values of $\epsilon_{r}$, coupled to a higher $\epsilon_{i}$ [17,33]. Also, ITO carrier concentrations are typically observed in the range of 10${}^{20}$–10${}^{21}$ cm${}^{-3}$ [41,46], similarly to the findings presented herein. We note that varied angle spectroscopic ellipsometry (VASE) is an alternative and very robust, low-noise measurement technique, that has frequently been used for the determination of the properties of TCOs [47,48,49,50]. VASE, however, also requires a model with assumptions on material parameters, hence, many of the insights we provide here also apply there. In addition, VASE can provide an analysis of the graded nature of ITO, which our resonance technique cannot. However, for a device application, one is typically concerned with the effective index of the material, which our resonance technique readily provides. Our technique also highlights one of the issues with ENZ materials, namely their high losses; many authors aim to enhance light–matter interactions, often by resonance techniques; however, we show that the resonance quality factor and, especially amplitude, readily declines as the ENZ point is approached. This decline is also the reason for which we limited our investigation to the wavelength range of λ < 1 μm.

The body of this work purely focuses on ITO; however, due to the generality of this study, many of the findings can be applied to several other members of the TCO family. For example, materials such as aluminium zinc oxide (AZO) and gallium zinc oxide (GZO) are alternative TCOs commonly used in photonic semiconductors. The variation in the oxygen concentration during DC magnetron sputter-deposited ZnO films has also been shown to vary characteristics such as the crystallinity [51], with a reduced oxygen flow producing a more crystalline film [52]; a result that agrees with the study we present herein. Additionally, the $\epsilon_{\infty }$ of GZO is shown to vary with changes in deposition conditions [53], corroborating the results shown herein. Hence, by applying the model we detail here to other TCOs, a method of determining the Drude dispersion characteristics can be determined. It is also reasonable to extrapolate the oxygen variation during deposition effects regarding film crystallinity, and ultimately conductivity, to other TCO materials, as well as the optical results to any TCO materials that follow the Drude dispersion model. An additional application of this work lies in the use of ITO in hyperbolic metamaterials (HMMs). Such structures are capable of enhanced confinement, decay-rate engineering, and subwavelength imaging due to the highly anisotropic permittivity [54,55,56,57]. The findings presented here act as a solid foundation for understanding the ITO dispersive permittivity, permitting further studies in this research area.

The discussion so far has primarily focused on the linear optical properties of ITO. However, several of the relevant parameters, such as the electron effective mass, collision frequency, and carrier concentration, are also of interest for studying nonlinear effects. For example, ITO has been shown to exhibit an extremely large ultrafast nonlinearity at the ENZ point [58], providing an optically induced enhancement to changes in the refractive index. Moreover, surface plasmons have been observed on ITO-coated structures, producing a strong nonlinearity and a large refractive index change within a half-wavelength range [59]. Hence, the linear photonic characterisation of TCO dispersion trends and ENZ behaviour we detail here provide a fundamental basis for also exploring nonlinear optical responses of TCOs.

## 6. Conclusions

In summary, we report a comprehensive study of the photonic properties of ITO thin films deposited by DC magnetron sputtering. We find that oxygen flow during deposition is the key parameter for controlling the conductivity of the films and note that a 0% oxygen flow produces the highest conductivity of (1.3 ± 0.2) × 10${}^{3}$ S/cm. We measured the crystallinity of the films using X-ray diffraction, finding that crystallinity decreases with oxygen flow, which explains the changes in mobility and high-frequency permittivity, two parameters that are assumed by many authors to be constant. The optical properties of the films were assessed by fabricating guided mode resonance gratings; by matching the measured resonance wavelengths for both polarisations, the real part of the permittivity is accurately determined. The imaginary part is then extracted from the amplitude of the resonances. We discuss the effect of all material parameters on ITO dispersion and highlight the importance of experimentally determining these values, as opposed to using quoted literature values. Overall, by determining all of the relevant parameters and showing how they depend on deposition conditions, the study provides a much-needed reference to the research area. We hope that the work will guide future studies on novel applications of ITO, and TCOs in general, as photonic materials.

## Figures and Tables

**Table 1:** Average grain sizes for different crystal orientations for varying oxygen flows during the deposition of ITO films (for more detail on uncertainty analysis, see Supplementary Material, Part 8).

O${}_{2}$ Gas Flow (%)	Conductivity (S/cm)	Orientation	FWHM (°)	Average Grain Size (nm)
0	1260.90	(222) (440)	0.246 0.261	54.27
5	20.61	(222) (440)	0.291 0.313	45.69
20	56.92	(222) (440)	0.361 0.389	34.49

**Table 2:** Table showing Drude fit parameters for films with 0, 5, 20 and 27% oxygen flow during deposition (for more details on uncertainty analysis, see Supplementary Material, Part 8).

O${}_{2}$ Gas Flow (%)	0	5	20	27
Sheet Resistance (/Sq)	38.4	1935.2	714.2	1117.6
Conductivity, σ (S/cm)	1260.9	20.6	56.9	36.2
Carrier Density, N (cm${}^{-}$${}^{3}$)	2.77 × 10${}^{20}$	4.53 × 10${}^{18}$	1.25 × 10${}^{19}$	7.96 × 10${}^{18}$
Electron Mobility, μ (cm${}^{2}$/Vs)	28.43	24.30	1.11	1.35
Plasma Frequency, $\omega_{p}$ (rad·THz)	1376	616	1351	949
Electron Effective Mass, m${}_{e}^{*}$ (kg)	0.51 m${}_{e}$	0.42 m${}_{e}$	0.50 m${}_{e}$	0.50 m${}_{e}$
High-Frequency Permittivity, $\epsilon_{\infty }$	4.31	5.30	5.42	5.33
Collision Frequency, Γ (THz)	147	993	390	1478

## Data Availability

The data presented in this study are available upon request from the corresponding authors. Details on several repositories containing the computational analysis scripts are available in the Supplementary Material.

## Supplementary Materials

The following supporting information can be downloaded at: https://www.mdpi.com/article/10.3390/nano13131990/s1, Figure S1: Electron Beam Lithography Patterning Process; Figures S2 and S3: Atomic Force Microscopy Analysis; Figure S4: Fourier transform SEM analysis; Table S1: Anneal Parameter Variation; Figure S5: Optical Measurement Setup; Figure S6: Automated RCWA Analysis; Figure S7: Drude Regression Fit Analysis; Tables S2 and S3: Uncertainty Analysis; Figure S8: GMR Effective Bandwidth. References [60,61,62,63,64,65,66,67,68,69] are cited in the Supplementary Materials.
