# Supplementary material for: Photonic Characterisation of Indium Tin Oxide as a Function of Deposition Conditions
Source: Nanomaterials (Basel). 2023 Jun 30;13(13):1990. doi: 10.3390/nano13131990 (PMC10343510; doi:10.3390/nano13131990)
Supplement: Supplementary file 1 [file nanomaterials-13-01990-s001.zip › nanomaterials-2478422-supplementary.pdf]

Supplementary Materials: Photonic Characterisation of Indium Tin Oxide as a Function of Deposition Conditions

Samuel F. J. Blair *, Joshua S. Male *, Stuart A. Cavill, Christopher P. Reardon and Thomas F. Krauss

1. Device Fabrication & Measurement Techniques

1.1. Fabrication

Several fabrication steps are required for the production of the indium tin oxide (ITO) guided mode resonance (GMR) gratings. We start by cleaning 15x15 mm borofloat chips in Piranha solution (3 H_2SO_4 : 1 H_2O_2), followed by an acetone and isopropyl alcohol (IPA) rinse. For the ITO deposition, a 90/10 wt% In_2O_3/SnO_2 target was used in a DC magnetron sputtering system. A 20 standard cubic centimeters per minute (SCCM) Ar flow was used for all depositions, with the oxygen flow being injected directly into the gun. The working pressure is 7.5×10^{-3} mbar. Films were left to cool in vacuum overnight. We noticed that if films were exposed to air while hot, the conductivity decreases dramatically, which we assume is related to the incorporation of nitrogen. A film thickness of approximately 300 nm was used throughout the experimental campaign, which is necessary for obtaining high-quality guided mode resonances.

Samples were annealed in a cylindrical tube furnace with varying oxygen flows at a 5 °C/min ramp up to set temperatures, and down to 25 °C. We observed little change over the flow range, which suggests that the only requirement is to have sufficient oxygen available. Post-deposition film thicknesses were taken with a surface profilometer (Bruker DektakXT) on test samples.

Patterning was done on a Raith Voyager (R) electron-beam lithography tool, using a 40 µm medium current (0.6 nA) aperture and a 10 nm step size. Samples were spin coated in AR-P 13 at 5000 rpm for 60 seconds, followed by a 10-minute bake at 180 °C. Samples were then developed in Xylene for 2 minutes, followed by a rinse in IPA. Figure S1 describes the key steps of the fabrication process and shows an example GMR grating.

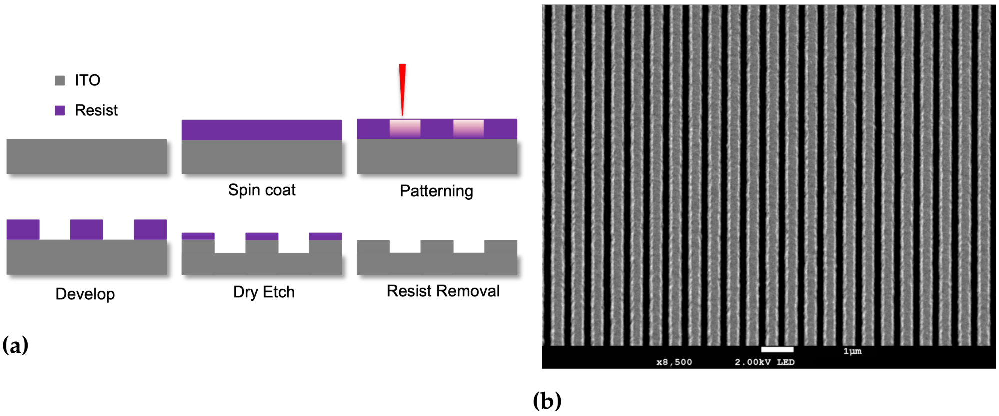

Figure S1. Patterning process using e-beam lithography.

Dry etching was performed in a reactive ion etching (RIE) tool, using a mixture of 21 Ar : 5 H_2 SCCM gas flow at a 3×10^{-2} mbar pressure and 450 V forward voltage. These conditions yield an etch rate of 5 nm/min and are a combination of the physical and chemical etching component of the Ar and the H_2, respectively. Regarding the chemical component, the H_2 reduces the metal oxide to form H_2O, which is volatile at the process pressure, whilst the Ar works to physically bombard the In and Sn. Samples were etched for 30 minutes, creating an etch depth of approximately 150 nm. Following etching, the remaining resist was removed by a rinse in 1165 Solvent remover at $\sim$ 65 °C, followed by

acetone and IPA rinses. Finally, samples were placed in a Plasma Asher with O_2 plasma for 10 minutes.

The developed dry etch recipe produces a satisfactory etch rate, reaching the required etch depth, however, at the cost of some roughness at the top of the grating ridges, which is partly attributed to film roughness (see Section 2, Atomic Force Microscopy). This approach could be improved by using a wet etch [1] with Oxalic acid, however, minimum feature sizes of only ∼2µm are achievable [2] at the same 5 nm/min rate as demonstrated above [3]. A preferred method would be the introduction of either CH_4 [4,5] or Cl_2 [6,7] gas with H_2 or Ar, however, these gases were not available at the time of the study. The introduction of either CH_4 or Cl_2 would likely increase the etch rate, reducing the etch time and removing the possibility of any damage to the film surface/grating ridges. All structures and films undertook all fabrication steps simultaneously, enabling a large degree of accuracy and reliability. A slight error in feature size may be introduced from sample to sample, however, we feel this error is negligible and is absorbed by the errors on the film's electrical parameters and optical spectroscopy data.

1.2. Measurement Techniques

1.2.1. X-ray diffraction (XRD)

XRD measurements were taken using a Rigaku SmartLab Automated XRD, using a Cu K alpha source at a wavelength of 15.406 nm.

1.2.2. Four Point Probe

The four-point probe measurements were taken on a Jandel RM3000+ test unit with a Jandel Cylindrical Four Point Probe. Multiple measurements were taken for each sample for error reduction.

1.2.3. Hall Probe

Hall probe measurements were taken using an Ecopia HMS-3000 Hall Measurement System using the Van der Pauw contact method [8], at a magnetic field strength of 0.56 T. Data was taken at various currents for each chip, with a Hall coefficient extracted from a tV vs IB_Z plot. Material parameters were then extracted using the following equations.

$$R_H = \frac{tV}{IB_Z} \quad (1)$$

$$R_H = -\frac{1}{eN} \quad (2)$$

$$\sigma = e\mu N \quad (3)$$

$$N_s = N_b t \quad (4)$$

Here, R_H is the Hall coefficient, t the film thickness, V the voltage between respective contact corners, I the operating current, B_Z the magnetic field, e the electron charge, N the carrier density, σ the conductivity, µ electron mobility, N_s the sheet carrier density in cm^{-2} and N_b the bulk carrier density in cm^{-3}.

2. Atomic Force Microscopy

Following grating etching, several analysis steps were undertaken to verify the grating parameters. To measure the etch depth, Atomic Force Microscopy (AFM) measurements were conducted on a Bruker BioScope Resolve AFM. A 2x2 µm scan size was used with 512 lines per scan at a 0.999 Hz scan frequency, using a Bruker RTESPA300 AFM tip. From each scan, the etch depth is easily determined, along with estimates of the period and filling factor of the gratings. As is apparent from the flat etch floor seen in Figure S2b, the AFM tip is of a sufficiently high aspect ratio to reach into the bottom of the structure. The period

was extracted from average distances between ridges in a given film, and the fill factor was taken from lines at the center of the sidewalls to account for the tip circumference during measurements. This data was then fed into a Rigorous Coupled Wave Analysis (RCWA) model to allow for a realistic simulation of the structures.

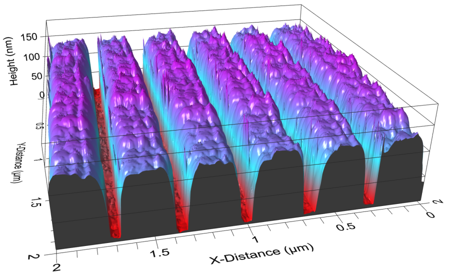

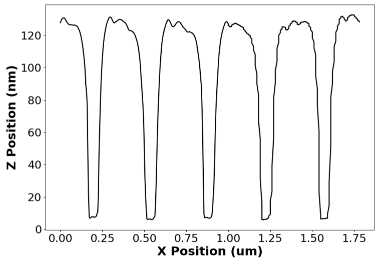

(b)

(a)

Figure S2. (a) Example AFM Scan across GMR grating, (b) example step data across the grating profile.

Additionally, it is worth investigating the film surface roughness before and after etching to analyze the effect of the developed etch on scattering loss. Figure S3 shows AFM micrographs before and after Reactive Ion Etching of an annealed and unstructured ITO surface, deposited with a 0% O_2 flow during deposition. Post deposition, the ITO film exhibits a relatively rough surface, reaching an average Root-mean-squared (RMS) surface roughness value of 2.09 ± 0.02 nm. This is in comparison to post-etch, where an average RMS surface roughness of 3.35 ± 0.16 nm was measured. Hence, a slight increase in the roughness is observed from the etch, which will increase the scattering losses observed in the optical results. Nonetheless, the increase is of a small magnitude, meaning the etch has not had a significant effect on the surface. In comparison, commercially available ITO is quoted to have a surface roughness of 1.8 nm RMS [9].

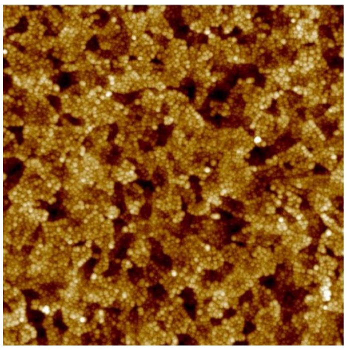

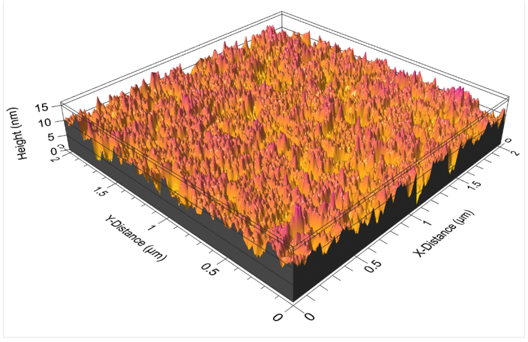

(a) **(b)**

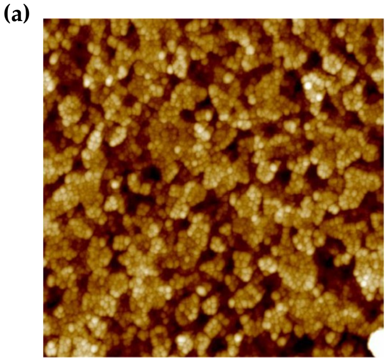

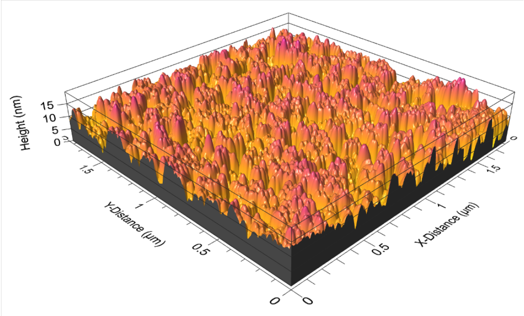

(c) **(d)**

Figure S3. 2D and 3D AFM scans of an ITO film deposited at 0% oxygen concentration during deposition before (a-b) and after (c-d) Reactive Ion Etching.

3. Fourier Transform Scanning Electron Microscope Image Analysis

To obtain a better value for grating parameters and to complement the AFM measurements, a Fourier transform-based scanning electron microscopy-image analysis process was developed, using a JEOL JSM-7800F Prime scanning electron microscope (SEM).

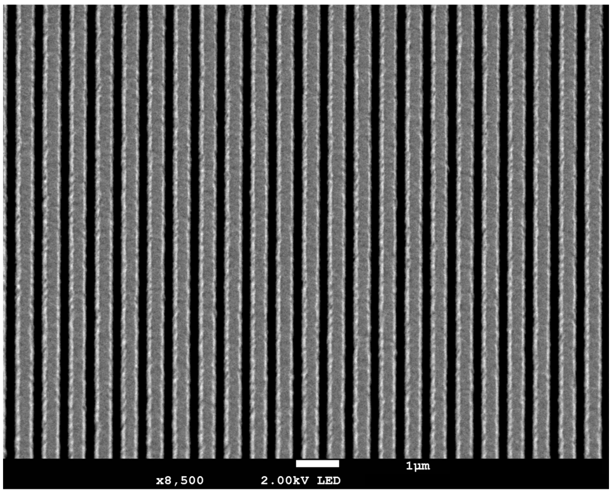

Figure S4. Example SEM micrograph of the fabricated ITO grating.

The SEM produces a grey-scale image of the fabricated gratings; by setting appropriate threshold values, a binary black-and-white picture is produced. A Fourier Transform then finds the dominant frequency component, i.e. the period. Using the Fourier Transform has the advantage that it finds the global period rather than the local period, which is subject to roughness.

4. Anneal Parameter Variation

While the oxygen flow rate during deposition turned out to be the most critical parameter in our process, the oxygen content during annealing does not seem to matter much. In order to verify this observation, we varied anneal temperature, time and gas flow rate. The results are shown in Table S1 for an array of 0% O_2 flow during deposition samples.

Table S1. Table showing sheet resistance as a function of annealing conditions for films deposited with 0% Oxygen.

Gas Flow (SCCM)	Anneal Temperature (°C)	Anneal Time (h)	Sheet Resistance (Ω/Sq)
Air	500	1	48.3 ± 0.5
500 O_2	500	1	41.7 ± 0.4
1000 O_2	500	1	38.9 ± 0.1
1000 O_2	600	1	59.6 ± 0.9
500 O_2	500	2	46.6 ± 0.4
500 O_2	300	1	88.9 ± 0.3

It is clear that the variation between the majority of the annealing conditions is small, as long as the temperature is at 500 °C or above; neither flow nor time have much further influence. Note that the remaining variations are much smaller than the variations seen with oxygen during deposition, as shown in Figure 2 of the main paper.

5. Reflectance Measurements

The experimental setup used to determine the GMR reflectance spectra is shown in Figure S5. A beam from a halogen lamp passes a polarizer and a Köhler lens to focus the beam onto the back focal plane of the objective, which yields collimated, polarised illumination of the grating. The reflected signal is collected by the 4x objective and focused into a spectrometer. The measured wavelength-resolved reflectance is then normalized relative to a mirror to obtain a background spectrum. A CCD camera is added for alignment purposes.

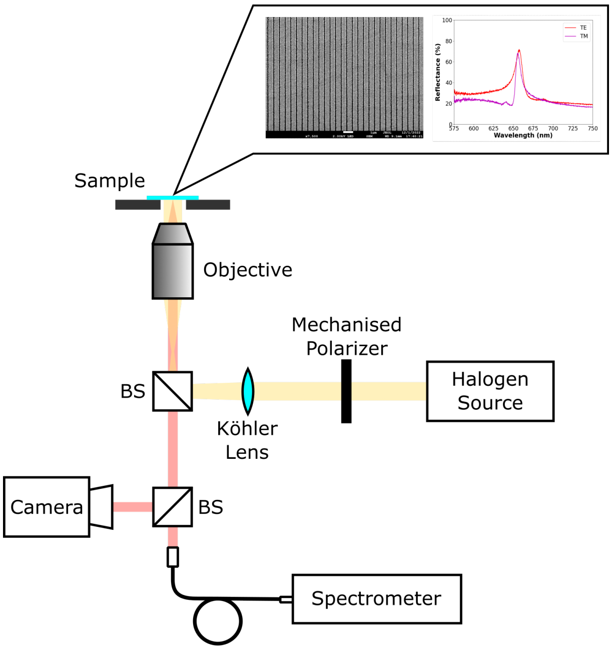

Figure S5. Optical set up for measuring the reflectance of guided mode resonance spectra for 1D gratings.

6. Rigorous Coupled Wave Analysis

Having determined the physical properties and TE and TM optical resonances for each grating, we used an RCWA software to link the real and imaginary permittivity to the observed resonances. Specifically, we used the Stanford Stratified Structure Solver (S4), which is a frequency domain linear Maxwell equation solver for layered periodic structures [10]. S4 computes the transmission and reflection spectra of periodic structures, which are then matched to experimentally obtained resonance peaks.

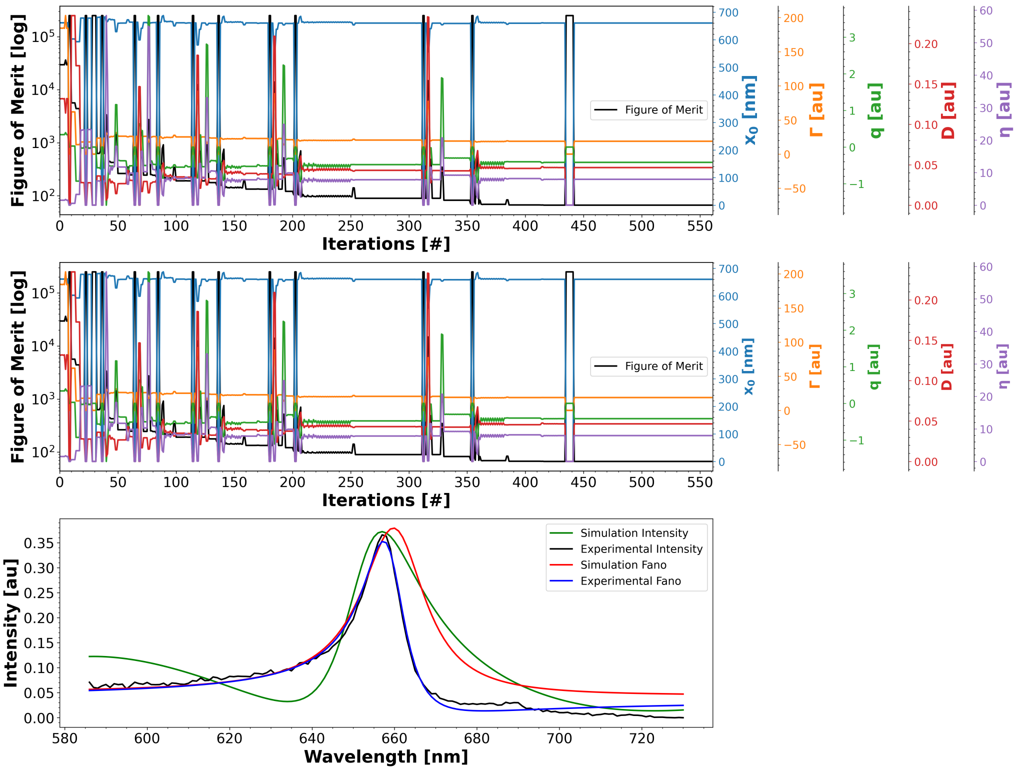

Figure S6. Example of an optimized RCWA analysis of resonant periodic structures in ITO.

7. Drude Regression Fit

Once the physical and optical parameters of the grating have been determined, the material parameters can be found from the Drude equation (equation (1) in the main text). These parameters, specifically the high-frequency permittivity, electron mobility, effective mass, and collision frequency can all be determined from a non-linear curve regression tool. We start by plotting the experimentally determined parameters, such as the permittivity at the grating resonance frequencies, which then act as known points of a non-linear regression. The unknown parameters are then given reasonable ranges within which they can vary without being restrictive, i.e. without biasing the routine towards an expected answer. An example real and imaginary dispersion plot is shown in Figure S7. Further details of the analysis are detailed in the software documentation.

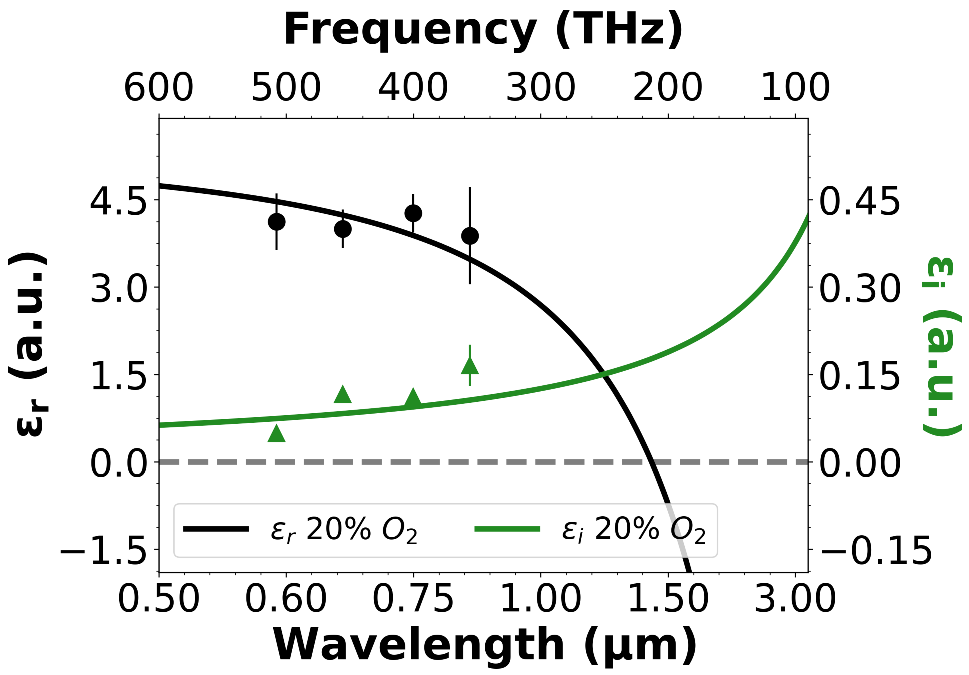

Figure S7. Example of optimized RCWA analysis of resonant periodic structures in ITO.

8. Uncertainty Analysis

The parameters from Tables 1 and 2 in the main text are shown below in Table S2 and S3 with associated errors.

Table S2. Average grain sizes for different crystal orientations for varying oxygen flows during the deposition of ITO films with associated errors.

O_2 Gas Flow (%)	Conductivity (S/cm)	Orientation	FWHM (°)	Average Grain Size (nm)
0	1260.90 ± 168.64	(222)<br>(440)	0.246 ± 0.001<br>0.261 ± 0.006	54.27 ± 0.92
5	20.61 ± 1.17	(222)<br>(440)	0.291 ± 0.016<br>0.313 ± 0.002	45.69 ± 0.45
20	56.92 ± 4.72	(222)<br>(440)	0.361 ± 0.007<br>0.389 ± 0.009	34.49 ± 0.91

Table S3. Table showing Drude fit parameters for films with 0, 5, 20 and 27% oxygen flow during deposition with associated errors.

**O_2 Gas Flow (%)**	**0**	**5**	**20**	**27**
Sheet Resistance (Ω/Sq)	38.4 ± 0.5	1935.2 ± 32.3	714.2 ± 31.0	1117.6 ± 19.7
Conductivity, σ (S/cm)	1260.9 ± 168.6	20.6 ± 1.1	56.9 ± 4.7	36.2 ± 2.9
Carrier Density, N (cm^{-3})	(2.77±0.73) × 10^{20}	(4.53±0.24) × 10^{18}	(1.25±0.62) × 10^{19}	(7.96±0.86) × 10^{18}
Electron Mobility, μ (cm^2/Vs)	28.43 ± 2.31	24.30 ± 2.52	1.11 ± 0.72	1.35 ± 0.94
Plasma Frequency, ω_p (rad·THz)	1376 ± 10	616 ± 10	1351 ± 10	949 ± 10
Electron Effective Mass, m_e^* (kg)	(0.51±0.18) m_e	(0.42±0.11) m_e	(0.50±0.35) m_e	(0.50±0.28) m_e
High Frequency Permittivity, ϵ_∞	4.31 ± 0.42	5.30 ± 0.46	5.42 ± 0.57	5.33 ± 0.43
Collision Frequency, Γ (THz)	147 ± 43	993 ± 77	390 ± 52	1478 ± 199

9. GMR Effective Bandwidth

Refractive index and loss data were recorded for each oxygen condition at resonant wavelengths in a 0.6-0.85 μm range (Figure 5a-d in the main text). The blue highlighted region in Figure S8a shows the 0.38-0.6 μm wavelength range. Here, dispersion curves for all conditions tended to saturate when approaching the high-frequency permittivity (y-axis intercept), displaying a flat horizontal dispersion trend. Hence, taking measurements in this region is arguably redundant. Moreover, to experimentally determine GMR resonances in this wavelength range, grating structures are required to be exceptionally small in some instances (period < 250 nm), presenting unnecessary challenges in the fabrication process for little gain in understanding the dispersion trend.

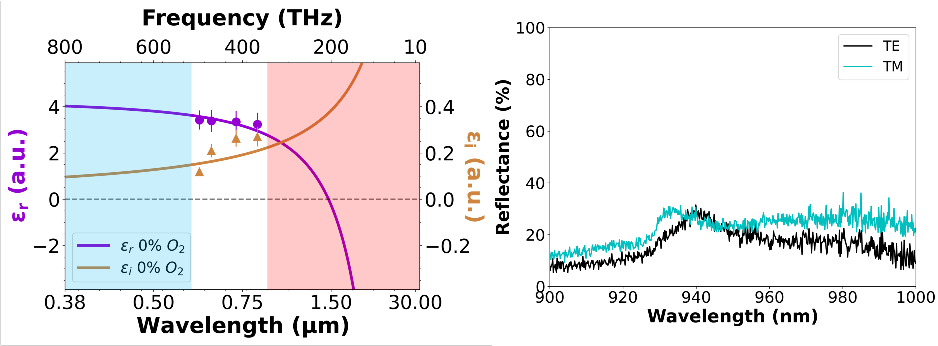

(a) (b)

Figure S8. (a) Example real and imaginary dispersion curves as seen in Figure 5a in the main text. The highlighted areas represent the physically restricted areas where reflectance measurements were not taken. (b) Example TE and TM spectra for a GMR grating with resonances at approximately 0.94 μm.

The red region to the right of Figure S8a displays a high wavelength range of 0.85-30 μm. As the real permittivity decreases with increasing wavelength, the loss of the

material increases, appearing more metallic as ϵ_r approaches negative values. Hence, when working with a nanostructured device, such as a GMR grating, observing a resonance in the high wavelength regime proves difficult when considering both the large absorption loss as well as the constant scattering effects. This intrinsic loss increase at larger wavelengths can be seen by the brown curve in Figure S8a. An exemplar spectrum is shown in Figure S8b of a grating resonant at approximately 0.94 µm for both polarizations. With the loss being large, the amplitude of the resonances is low, with a broadened Q-factor. As such, Fano fitting and extracting data from this peak introduces a degree of ambiguity and a large error in the measurement of the refractive index and loss data. Although measuring refractive index and absorption loss with the above method is loss-limited, extracting data from an actual device allows the full effective loss profile to be studied, as well as factoring in fabrication and equipment tolerances.

10. Github Repositories

We have made our code used for analysis and parameter extraction public in several Github repositories. These involve aspects such as Surface Profile Analysis, Microscopy Period analysis, an S4 Grating Optimiser and Drude Model Analysis. Repository links are listed below:

- https://github.com/jm1261/SurfaceProfileAnalysis
- https://github.com/jm1261/MicroscopyPeriodAnalysis
- https://github.com/jm1261/S4GratingOptimizer
- https://github.com/jm1261/DrudeModulators

References

1. Tsai, T.H.; Wu, Y.F. Wet etching mechanisms of ITO films in oxalic acid. *Microelectron. Eng.* **2006**, *83*, 536–541. [CrossRef]
2. Breen, T.L.; Fryer, P.M.; Nunes, R.W.; Rothwell, M.E. Patterning Indium Tin Oxide and Indium Zinc Oxide Using Microcontact Printing and Wet Etching. *Langmuir* **2002**, *18*, 194–197. [CrossRef]
3. Benor, A.; Gedifew, A.; Yigizaw, S.; Davis, K. Patterning Indium Tin Oxide Using Self-Assembled Monolayers as Etch Resists for Photovoltaic and Display Devices. *ACS Appl. Nano Mater.* **2022**, *5*, 6505–6512. [CrossRef]
4. Meziani, T.; Colpo, P.; Lambertini, V.; Ceccone, G.; Rossi, F. Dry etching of ITO by magnetic pole enhanced inductively coupled plasma for display and biosensing devices. *Appl. Surf. Sci.* **2006**, *252*, 3861–3870. [CrossRef]
5. Noemaun, A.; Mont, F.; Cho, J.; Schubert, E.; Kim, G.; Sone, C. Inductively coupled plasma etching of graded-refractive-index layers of TiO2 and SiO2 using an ITO hard mask. *J. Vac. Sci. Technol. Vacuum, Surfaces Film.* **2011**, *29*, 051302. [CrossRef]
6. Yokoyama, M.; Li, J.W.; Su, S.H.S.S.H.; Su, Y.K.S.Y.K. Characteristics of Indium-Tin Oxide Thin Film Etched by Reactive Ion Etching. *Jpn. J. Appl. Phys.* **1994**, *33*, 7057. [CrossRef]
7. Joo, Y.H.; Woo, J.C.; Choi, K.R.; Kim, H.S.; Wi, J.H.; Kim, C.I. Dry Etching of ITO Thin Films by the Addition of Gases in Cl_2/BCl_3 Inductivity Coupled Plasma. *Trans. Electr. Electron. Mater.* **2012**, *13*, 157–161. [CrossRef]
8. Ramadan, A.A.; Gould, R.D.; Ashour, A. On the Van der Pauw method of resistivity measurements. *Thin Solid Film.* **1994**, *239*, 272–275. [CrossRef]
9. Ossilla. ITO Glass Substrates. (Unpatterned).
10. Liu, V.; Fan, S. S4: A free electromagnetic solver for layered periodic structures. *Comput. Phys. Commun.* **2012**, *183*, 2233–2244. [CrossRef]
